# Whole-Exome Sequencing Identified a Novel DYRK1A Variant in a Patient With Intellectual Developmental Disorder, Autosomal Dominant 7

**DOI:** 10.7759/cureus.33379

**Published:** 2023-01-05

**Authors:** Koji Obara, Erika Abe, Itaru Toyoshima

**Affiliations:** 1 Department of Neurology, National Hospital Organization Akita National Hospital, Yurihonjo, JPN

**Keywords:** whole-exome sequencing, novel variant, mrd7, face2gene, dyrk1a

## Abstract

Intellectual developmental disorder, autosomal dominant 7 (MRD7; OMIM 614104) is a rare disease characterized by microcephaly, intellectual disability, speech delay, feeding difficulties, and facial dysmorphisms. This disorder is caused by pathogenic/likely pathogenic variants of the *DYRK1A* gene, which encodes dual-specificity tyrosine-phosphorylation-regulated kinase 1A. Here, we report a case of MRD7 that was diagnosed using Face2Gene and whole-exome sequencing (WES). A 22-year-old man presented with microcephaly, intellectual disability, slender body, long slender fingers, and facial dysmorphisms. He was previously diagnosed with Cornelia de Lange syndrome (CdLS) at four years of age. However, his CdLS clinical diagnostic score was low at 22 years of age. The Face2Gene application introduced several candidate diseases including MRD7. Finally, by utilizing WES and Sanger sequencing analysis of cloned cDNA, we identified a novel heterozygous duplication variant (c.848dup, p.(Asn283LysfsTer6)) in the *DYRK1A* gene, which introduces a premature stop codon. This report provides more information about the phenotypic spectrum of a young adult patient with MRD7. Face2Gene helped us introduce candidate diseases of the patient. Registering further genetically confirmed cases with MRD7 will improve the accuracy of the diagnostic recommendations in Face2Gene. Moreover, WES is a powerful tool for diagnosing rare genetic diseases, such as MRD7.

## Introduction

Intellectual developmental disorder, autosomal dominant 7 (MRD7; OMIM 614104), also known as DYRK1A (haploinsufficiency) syndrome, is a rare autosomal dominant disease with a prevalence of less than 1/1,000,000 [1-3]. MRD7 is characterized by microcephaly, intellectual disability, speech delay, feeding difficulties, and distinct facial dysmorphisms, including prominent ears, deep-set eyes, mild upslanted palpebral fissures, a short nose with a broad nasal tip, and retrognathia [1,4,5]. Pathogenic/likely pathogenic variants of the *DYRK1A* gene, which encodes dual-specificity tyrosine-phosphorylation-regulated kinase 1A (DYRK1A), are responsible for MRD7. DYRK1A has a serine/threonine kinase with numerous proteins involved in cell proliferation, neuronal morphogenesis, synaptogenesis, and synaptic function, and it is essential for brain development and function [4,6,7]. The *DYRK1A* gene is located in chromosome 21q22.13, which is also the Down syndrome (DS) critical region [7,8]. The overexpression of DYRK1A produces intellectual disabilities in DS. Contrarily, haploinsufficiency of DYRK1A through a heterozygous chromosomal loss or intragenic variants causes MRD7 [7,8], suggesting that DYRK1A influences the cognitive function in a dose-dependent manner [2]. Herein, we report a case of MRD7 in whom Face2Gene introduced several genetic syndromes, including MRD7, as candidates. We also identified and confirmed a de novo novel variant in the *DYRK1A* gene of the patient by whole-exome sequencing (WES).

## Case presentation

**Figure 1:**
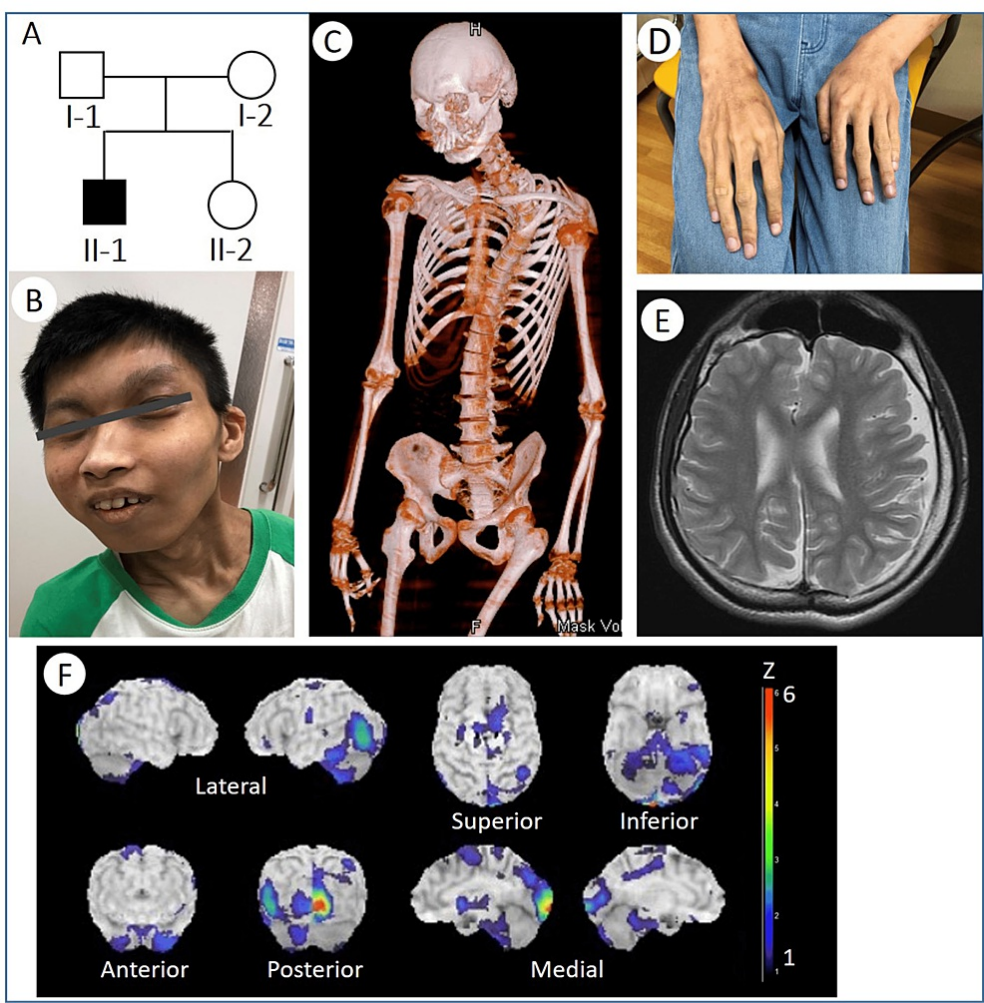
Clinical findings (A) Pedigree chart of the patient’s family. All family members underwent DNA analyses. (B) The face of a patient with microcephaly, thick eyebrows, long eyelashes, deep-set eyes, right ptosis, mildly upslanted palpebral fissures, broad nasal tip, prominent nasal bridge, low-set and malformed ears, micrognathia, and overjet. (C) Three-dimensional reconstruction image of a bone computed tomography scan shows pectus excavatum and scoliosis. (D) Long slender fingers (arachnodactyly). (E) Brain magnetic resonance imaging reveals diffuse and mild cortical atrophy and right-sided deviation of the cerebrum. (F) Images of cerebral blood flow with single-photon emission CT using N-isopropyl-p-[^123^I] iodoamphetamine (IMP-SPECT). The Z-score shifts to red (6) as the degree of the decrease in the cerebral blood flow that is larger than that of an age-matched normal database. IMP-SPECT reveals a decreased blood flow in the right medial occipital cortex, left lateral occipital cortex, and bilateral anterior and left infero-posterior cerebellum.

The patient is a 22-year-old man who was born to healthy non-consanguineous parents. His parents and sister are alive and well without any dysmorphism (Figure 1A). The mother’s pregnancy and delivery of the patient were normal. His birth weight was 2.918 g, birth length was 46 cm, and head circumference was 31 cm. From birth onward, he had feeding problems including poor suckling. His height and weight were below −2 standard deviation until at least 10 years of age. At 10 months of age, he experienced his first seizure as febrile convulsions, which often appeared until his early teens. He sat without any support at 13 months and walked at 20 months. During infancy and childhood, he had difficulty falling asleep. At four years of age, he was clinically diagnosed with Cornelia de Lange syndrome (CdLS; OMIM 122470, 300590, 300882, 610759, and 614701) due to his facial dysmorphism, microcephaly, short stature, and intellectual disability. At seven years of age, he was only able to pronounce a couple of simple syllables; subsequently, he did not acquire any more words. At approximately age 15, he became taller and noticeably slender. Concurrently, the frequency of his febrile convulsions significantly decreased. At the age of 19 years, he underwent two surgeries for spontaneous pneumothorax caused by ruptured pulmonary bullae. At the present age of 22, his height was 157.2 cm, body weight was 38.9 kg, and head circumference was 51 cm. He had dysmorphic facial features (Figure 1B), including thick eyebrows, long eyelashes, deep-set eyes, right ptosis, mild upslanted palpebral fissures, broad nasal tip, prominent nasal bridge, low-set and malformed ears, micrognathia, and overjet. Further, he had a slender body with pectus excavatum, scoliosis (Figure 1C), and long slender fingers (arachnodactyly) (Figure 1D). He was hyperactive with friendly behavior. Contrarily, he did not have anxious or stereotypic behaviors, including self-injury. While he could only speak a few words, such as “papa” and “mama,” he was able to obey some basic verbal instructions, such as “sit” and “stand.” Additionally, his head showed a right-side rotation, and his neck bent forward. He had mild hypertonus in the extremities and limited extension of the elbow and knee joints by contractures. His muscle stretch reflexes were bilaterally hyperactive in the upper and lower limbs, and the plantar flexor response was observed bilaterally. He could run, albeit in small steps. The results of the complete blood count, liver and renal function tests, thyroid hormone, uric acid, plasma ammonia, and vitamin B_12_ were all within normal limits. His karyotype was 46, XY. Electrocardiogram and echocardiography showed no abnormal findings. Brain magnetic resonance imaging revealed diffuse and mild cortical atrophy and right-sided deviation of the cerebrum (Figure 1E). Single-photon emission computed tomography using N-isopropyl-p-[^123^I] iodoamphetamine (IMP-SPECT) revealed hypoperfusion in the right medial occipital cortex, left lateral occipital cortex, and bilateral anterior and left infero-posterior cerebellum (Figure 1F). According to the criteria proposed in the first international consensus statement of CdLS, the patient’s clinical score was 5 (total score of 19), including one cardinal feature (thick eyebrows), which is sufficient to warrant molecular testing for CdLS [9].

We uploaded the patient’s frontal facial photograph and relevant clinical features to the Face2Gene (FDNA Inc, Boston, USA) application. As a result, Face2Gene introduced the following candidate diseases: Angelman syndrome (AS, OMIM 105833), Witteveen-Kolk syndrome (WKOS; MIM 613406), Rubinstein-Taybi syndrome (RTS; OMIM 180849), and MRD7.

We then applied fluorescence in situ hybridization analysis with the D15S10 probe (BML Inc, Saitama, Japan), which did not detect the 15q11.2-q13 deletion associated with AS.

Next, we applied the following methods to confirm the patient's genetic diagnosis. Genomic DNA of the quartet (the patient and his unaffected parents and sister) was extracted from the peripheral blood using standard methods for whole-exome sequencing (WES) provided by Veritas Genetics Asia Inc. (China). Then, 200 ng of genomic DNA was sheared to 150 bp target DNA fragments for library preparation, which were constructed with pre-capture library, library hybridization, and capture with SureSelect V6 probes (Agilent, USA), post capture library amplification, and purification using the SureSelect XT HS Target Enrichment System (Agilent, USA). After purification, the indexed libraries were quantified and qualified using Qubit 2.0 Fluorometer (Thermo Fisher) and Qsep100 [Bioptic, Taiwan (R.O.C.)]. All samples were diluted and pooled for multiplexed sequencing on NovaSeq 6000 (Illumina, USA). Bioinformatic analysis was performed by cBioinformatics, Inc. (Tokyo, Japan). Reads were aligned to the human genome assembly GRCh38/hg38 using Burrows-Wheeler Aligner Enrichment (v0.7.17) [10]. The Genome Analysis Toolkit (GATK v4.1.9. 0) was used for genotyping, indel discovery, single-nucleotide variants, and de novo variant calling, as well as for indel realignment and base quality score recalibration. Detected variants were annotated using SnpSift (v5.0c) and SnpEff (v5.1) [11-13]. Filtering of candidate variants was subsequently performed as follows. First, variants with an allele frequency >0.01% in the jMorp, 14KJPN (https://jmorp.megabank.tohoku.ac.jp/202206/), and 1000 Genomes and gnomAD v2 database in East Asian populations were removed. Second, only de novo variants with high confidence were extracted. Third, silent variants that did not affect protein sequence were removed. As a result of these filters, we narrowed it down to 13 variants. Among genes containing these variants, GeneReviews® (https://www.ncbi.nlm.nih.gov/books/NBK1116/) listed the following five genes: *TNN*, *SEDT2*, *NADK2*, *ZNF462*, *CACNB2*, and *DYRK1A* (accessed: December 24, 2022). Finally, among the genes for which variant was detected, the *DYRK1A* gene was proposed as the responsible gene based on the quality of variant calling, such as allele depth, genotype quality, effect of protein function, and similarity of the clinical findings with previous reports. Next, mRNA was purified according to the manufacturer’s protocols (Nippon Gene, Japan), and cDNA was obtained via reverse transcription using the Clone Avian Myeloblastosis Virus First-Strand cDNA Synthesis Kit (Invitrogen, USA). The proposed region in exon 7 of the *DYRK1A* gene in the patient, his parents, and sister was amplified via polymerase chain reaction (PCR) (SimpliAmp Thermal Cycler) and cloned using the Zero Blunt PCR Cloning Kit (Invitrogen). Sanger sequencing was performed on the obtained multiple clones, with the result consistent with the variant proposed by WES.

**Figure 2:**
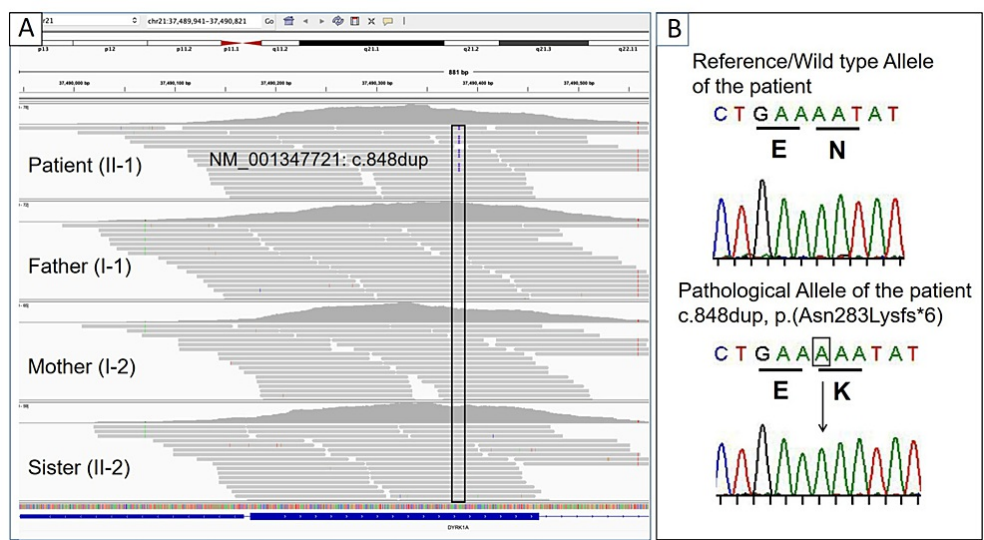
Genetic testing (A) Integrative Genomics Viewer shows NM_001347721 (*DYRK1A*):c.848dup pathogenic variant in the patient. His parents and sister did not carry this variant, indicating a de novo status. (B) Sanger sequencing analysis of cloned cDNA from the patient’s white blood cell. The results indicate a heterozygous duplication variant (box and black arrow) in exon 7 of the *DYRK1A* gene [c.848dup, p.(Asn283Lysfs*6)], introducing an early stop codon at six amino acids downstream from the altered codon.

As a result of the abovementioned analysis, the patient showed a novel heterozygous duplication variant (NM_001347721: c.848dup, p.(Asn283LysfsTer6)) in the coding region of exon 7 of the *DYRK1A* gene (Figure 2). Given that his parents did not carry the variant, it was classified as a de novo variant. To date, this variant has not been cited in gnomAD (https://gnomad.broadinstitute.org/), Clinvar (http://www.ncbi.nlm.nih.gov/clinvar), iJGVD (https://jmorp.megabank.tohoku.ac.jp/ijgvd/), and HGMD (http://www.hgmd.cf.ac.uk/ac/index.php). This variant was classified as being pathogenic according to the American College of Medical Genetics and Genomics criteria [14]. In this WES analysis, we did not define any pathogenic/likely pathogenic variants in the coding regions of the genes associated with CdLS (*NIPBL*, *SMC1A*, *SMC3*, *RAD21*, *HDAC8*, and *ANKRD11*), AS (*UBE3A*), WKOS (*SIN3A*), and RTS (*CREBBP* and *EP300*).

## Discussion

We identified a novel de novo heterozygous variant in the *DYRK1A* gene, i.e., c.848dup, p.(Asn283Lysfs*6) in our patient and diagnosed him with MRD7. The variants associated with MRD7 are widely distributed in the *DYRK1A* gene [3]. Many of these variants introduce a premature stop codon via nonsense, frameshift, and splice site variants, leading to a loss of function [3]. Contrarily, several missense variants associated with MRD7 have been reported, primarily located in the kinase domain encoded by the *DYRK1A* gene, and affecting its catalytic function [15]. The variant in our patient is located within the kinase domain and causes premature truncation of the DYRK1A protein, impairs kinase activity, and is responsible for the patient's phenotype. All the variants in the *DYRK1A* gene in MRD7 are heterozygous, which leads to haploinsufficiency for the kinase activity.

Our patient had thick eyebrows and long eyelashes, similar to a patient with CdLS, but he had a slender body, long slender fingers, and friendly behavior, unlike patients with CdLS [9].

In the preliminary step of performing WES, we used the Face2Gene application, which introduced the following candidate diseases; AS, WKOS, RTS, and MRD7. These diseases and CdLS mostly have autosomal dominant inheritance, caused by de novo pathogenic variants, and share the following phenotypes: intellectual disability, facial dysmorphisms, speech delay/absence, behavior issues, microcephaly, feeding difficulty, sleep disorder, seizure, eye involvements such as strabismus, and skeletal anomalies (Table 1) [1,9,16-18].

**Table 1:** Differences in clinical features between intellectual developmental disorder, autosomal dominant 7 (MRD7) and its related diseases Adapted with the information from GeneReviews® (https://www.ncbi.nlm.nih.gov/books/NBK1116/) and Orphanet (https://www.orpha.net/consor/cgi-bin/index.php) included. AD, autosomal dominant; ADHD, attention deficit hyperactivity disorder; AS, Angelman syndrome; ASD, autism spectrum disorder; CdLS, Cornelia de Lange syndrome; GERD, gastro-esophageal reflux; MRD7, Intellectual developmental disorder, autosomal dominant 7; OAS, obstructive sleep apnea; RTS, Rubinstein–Taybi syndrome; WKOS, Witteveen–Kolk syndrome. *A deletion in the maternally inherited 15q11.2–q13 region (which includes the *UBE3A* gene), including uniparental disomy and an imprinting defect (>80%). A pathogenic variant in maternally inherited *UBE3A* (11%).

Diseases	MRD7	CdLS	AS	WKOS	RTS
Prevalence	1:500,000–1,000,000	1:10,000–100,000	1:12,000–24,000	<1:1,000,000	1:100,000–125,000
Gene (Inheritance)	DYRK1A (AD)	NIPBL (>80%, AD) Other genes (<10%)	UBE3A (*footnote)	SIN3A (AD)	CREBBP (50%–60%, AD) EP300 (8%–10%, AD)
Intellectual disability	+	+	+	+	+
Speech delay/absence	+	+	+	+	+
Behavior issue	+	+	+	+	+
Microcephaly	+	+	+	+	+
Feeding difficulty	+	+	+	+	+
Sleep disorders	+	+	+	+ (rare)	+ (OAS)
Seizure	+	+	+	+	+
Gait disorders	+	+	+	-	+
Eye involvements	+	+	+	+	+
Skeletal anomalies	+	+	+	+	+
Facial dysmorphisms	+	+	+	+	+
Visceral anomalies and others	Urogenital anomalies, slender body	Hearing loss, GERD, cryptorchidism, obesity		Hypotonia	Cardiac anomaly, renal anomaly, cryptorchidism, obesity

Contrarily, each disease has characteristic dysmorphic facial features (Table 2).

**Table 2:** Facial dysmorphisms in intellectual developmental disorder, autosomal dominant 7 (MRD7) and its related diseases Adapted with the information from GeneReviews® (https://www.ncbi.nlm.nih.gov/books/NBK1116/), Orphanet (https://www.orpha.net/consor/cgi-bin/index.php), and Human Phenotype Ontology (https://hpo.jax.org/app/) included. AS, Angelman syndrome; CdLS, Cornelia de Lange syndrome; MRD7, Intellectual developmental disorder, autosomal dominant 7; -, negative or not mentioned; RTS, Rubinstein–Taybi syndrome; WKOS, Witteveen–Kolk syndrome.

	This case	MRD7	CdLS	AS	WKOS	RTS
Narrow forehead	+	+	-	-	-	-
High forehead	-	-	-	-	+	-
Hypertelorism	-	-	-	-	+	+
Deep-set eye	+	+	-	+	+	-
Synophrys	+	-	+	-	-	-
Arched and/or thick eyebrows	+	-	+	-	+	+
Long eyelashes	+	-	+	-	-	-
Upslanted palpebral fissure	+	+	-	-	+	-
Down-slanted palpebral fissure	-	-	-	-	+	+
Short nose	-	+	+	-	+	-
Wide nasal bridge	+	+	-	-	+	+
Beak-shaped nose	-	-	-	-	-	+
Anteverted nares	-	-	+	-	+	-
Prominent ear	+	+	-	-	-	-
Long and/or smooth philtrum	-	-	+	-	+	-
Thin upper lip vermilion	-	-	+	-	+	-
Prominent chin	-	-	-	+	-	-
Wide mouth	-	-	-	+	+	-
Downturned corners of mouse	-	-	+	-	-	-
Protruding tongue	-	-	-	+	-	-
Wide-spaced teeth	-	-	-	+	-	-
High palate	-	-	+	-	+	+
Talon cusps	-	-	-	-	-	+
Retrognathia and/or micrognathia	+	+	+	-	+	+
Prognathia	-	-	-	+	-	-

MRD7 has facial features such as prominent ears, deep-set eyes, upslanted palpebral fissures, a short nose with a broad nasal tip, and retrognathia. Given that the reported prevalence of MRD7 is less than 1/1,000,000 [1,2], which is rare compared to 1:10,000 to 30,000 for CdLS [9], it is not easy for nonexpert clinicians to differentiate these syndromes based on facial dysmorphisms. Therefore, Face2Gene helped us introduce candidate diseases for the patient. Given that Face2Gene applies a deep learning algorithm, the more MRD7 cases during or after diagnosis are registered, the higher the accuracy.

In addition to the typical dysmorphic symptoms of MRD7, our patient presented with distinct long slender fingers (Figure 1D). To date, two reports describe tapered fingers in children with MRD7 [5,19]. The long slender fingers may become more pronounced or noticeable with age. Further case accumulation is needed to determine whether long slender fingers is a characteristic feature in adolescent and adult patients with MRD7.

Finally, by utilizing WES, variant of the *DYRK1A* gene was identified in our patient. Although WES remains expensive and its use in clinical practice is limited, its accessibility has expanded in recent years. Therefore, we believe that WES is a powerful and recommended tool for diagnosing rare genetic diseases from the initial steps.

## Conclusions

This report provides more information about the phenotypic spectrum of a young adult patient with MRD7. We identified a novel variant in the *DYRK1A* gene by utilizing WES. Face2Gene helped introduce candidate diseases for the patient. Registering further genetically confirmed cases with MRD7 will improve the accuracy of diagnostic recommendations in Face2Gene. WES is a powerful tool for diagnosing rare genetic diseases, such as MRD7.
